# Characterization of Nutritional, Antinutritional, and Mineral Contents of Thirty-Five Sorghum Varieties Grown in Ethiopia

**DOI:** 10.1155/2020/8243617

**Published:** 2020-03-11

**Authors:** Masresha Minuye Tasie, Belay Gezahegn Gebreyes

**Affiliations:** Food Science and Nutrition, Ethiopian Institute of Agricultural Research, P.O. Box 2003, Addis Ababa, Ethiopia

## Abstract

An experiment was carried out to characterize the proximate compositions and antinutritional and mineral contents of sorghum varieties released for production by the Ethiopian sorghum improvement programme. Sorghum is an extensively researched crop in Ethiopia. However, comprehensive information on nutritional, antinutritional, and mineral content has not been generated. In the present study, thirty-five sorghum varieties released by the national sorghum improvement programme were used and evaluated for their proximate compositions, tannin, and mineral nutrient. AOAC methods of analysis were used for proximate compositions and mineral content together, i.e., whereas for tannin, vanillin-HCL assay methods of analysis were used. Differences between sorghum varieties were significant (*P* ≤ 0.05) for all measured parameters. Proximate composition values such as moisture, ash, crude fat, crude fiber, crude protein, and CHO varied from 9.66 to 12.94, 1.12 to 2.29, 2.48 to 4.60, 2.17 to 8.59, 8.20 to 16.48, and 67.56 to 76.42, respectively. The highest mineral content of P (367.965), Na (6.151), Mg (207.526), K (314.011), Ca (67.159), Fe (14.018), and Zn (6.484) as measured by mg/100 g was found from the varieties Macia, Abshir, Chiro, Birmash, Dagem, and Assossa-1 (Fe and Zn), respectively. Maximum tannin values of 3337.200 and 2474.7 mg/100 g were obtained from Lalo and Dano, respectively. The varieties such as Miskir, Abshir, ESH-1, Meko-1, Red Swazi, and Karimtams have higher nutritional and mineral and lower antinutritional values among the tested varieties. The abovementioned varieties should be considered for food product development due to their nutritional qualities.

## 1. Introduction

Sorghum (*Sorghum bicolor* L. Moench) is the sixth most planted crop in the world, and it is one of the most important cereals used as a staple food for those primarily living in arid and semiarid areas [1]. In Africa, it is the second most important cereal in which around 300 million people depend on it as their daily consumptions (1). It is consumed mostly in northern China, India, and southern Russia, where about 85% of the crops are consumed directly as human food [2]. Sorghum has greater drought tolerance, soil toxicities, and temperature variation than other cereals and requires minimal fertilizers for cultivation, thus playing a critical role for food security in some semiarid areas of Asia, Africa, and Latin America [3]. It is considered one of the potential crops to alleviate the challenges of recurrent drought in Africa.

In Ethiopia, sorghum is the third (3^rd^) most important staple cereal crop after teff and maize [4]. Sorghum crop is grown in almost all regions of Ethiopia and used as a staple food crop on which the lives of millions of poor Ethiopians depend on it. Sorghum is used in various ways in our country; the grain is used for human foods such as injera, bread, porridge, Nifro, infant food, syrup, and local beverage known as “*Tella*” and “*Arekie*.” And also, the leaf and stalk are used for animal feed, and further, the stalks are used for the construction of houses and fences and as fuelwood. Melkassa center of the Ethiopian Institute of Agricultural Research has been working on the sorghum over the last many years; during this period, varieties of sorghum have been released. The released varieties had different specific qualities such as disease resistance, malt quality, and earliness. However, none of the varieties had no comprehensive nutritional profile data, since the quality of sorghum produced worldwide is affected by biotic and abiotic constraints [5]. Therefore, the aim of the present study was to profile the nutritional value, antinutritional value, and mineral contents of sorghum varieties grown in different agroecologies of Ethiopia. Knowing the sorghum nutritional diversity would have a direct impact on the improvement of sorghum for quality breeding and for food product development to reduce the prevalence of food insecurity, malnutrition, and mineral deficiency, especially for those low-income communities.

## 2. Materials and Methods

### 2.1. Materials

Studies were conducted in Melkassa Agricultural Research Center, which is one of the research centers of Ethiopian Institute of Agricultural Research (EIAR) found in the Ethiopian Rift Valley, 117 km away from Addis Ababa in the southeast direction located at 8024′N and 39012′E and an altitude of 1550 meters above sea level. The mean minimum and maximum temperatures of the environment are 13.8°C and 28.6°C, respectively. Andosol, which is of volcanic origin with pH value ranging from 7 to 8.2, is mostly the center soil type that the crop grow in. In this study, thirty-five Sorghum varieties obtained from the sorghum improvement programme based at the Melkassa center of EIAR were considered. Samples of this study were cleaned manually from dust particles, damaged seeds, and strange materials. Detail information about the selected sorghum grain is mentioned in Table 1. The facilities available in the food science lab of the Melkassa center of EIAR were used.

### 2.2. Methods of Analysis

#### 2.2.1. Nutritional Analysis

The representative samples of sorghum varieties moisture content, ash, fat, fiber, protein, total carbohydrate, food energy value, mineral content, and tannin were determined. All the determinations were done in triplicate, and the results were expressed as the average result of the value except for the mineral content.

*(1) Moisture Determination*. It is determined by the method of AOAC 925:10 [6]. Two grams (2 g) of well-homogenized sorghum flour samples was transferred to the dried and weighted dishes. Samples containing dishes were placed in the drying oven and dried for 1 h at 130°C/until the constant weight of the sample is maintained. Then, the dried samples were removed from the drying oven and then cooled in a desiccator at room temperature and reweighted.

(1) $$\begin{array}{c} \text{Moisture }\left(\%\right)=\frac{\left(W_{1}-W_{2}\right)*100}{S_{\text{W}}}, \end{array}$$

where *W*_1_ is the weight of the cap and fresh sample, *W*_2_ is the weight of the dry sample and cap, and *S*_W_ is the sample weight.

*(2) Ash Determination*. It is determined by methods of AOAC 923:03 [6]. Four (4) grams of well-homogenized sorghum flour was measured and put into a clean crucible of predetermined weight. The sample-containing crucible was placed in a muffle furnace at 550°C. The samples were ignited until light gray was obtained, and then, the sample was removed, cooled in a desiccator at room temperature, and weighted.

(2) $$\begin{array}{c} \text{Ash }\left(\%\right)=\frac{\left(W_{1}-W_{2}\right)*100}{S_{\text{W}}}, \end{array}$$

where *W*_1_ is the weight of the ash+crucible after ashing, *W*_2_ is the weight of the empty crucible, and *S*_W_ is the weight of the sample taken.

*(3) Fat Content Determination*. It was performed according to the method of AOAC 945:16 [6], Soxhlet extraction method. Two grams of sorghum flour was transferred into a previously prepared extraction thimble. The sample-containing thimble was plugged with fat-free absorbent cotton wool. The Soxhlet extraction apparatus was assembled and filled with petroleum ether spirit to a half capacity of the volume of the flask before the fat of the sample is extracted. Then, the extraction continued for 4 hours. After completing the time, the extracted fat was removed, and then, oil/fat-containing flasks were attached to the rotary evaporator to evaporate the major portion of the solvent. Using a dry oven to evaporate the last traces of the solvent at 103°C for 30 min, the flasks were later dried and cooled in a desiccator and reweighed.

(3) $$\begin{array}{c} \text{Fat }\left(\%\right)=\frac{\left(W_{\text{f}}-W\right)*100}{S_{\text{W}}}, \end{array}$$

where *W*_f_ is the weight of the receiver flask and fat deposit, *W* is the weight of the empty receiver flask only, and *S*_W_ is the weight of the sample used.

*(4) Crude Fiber Content Determination*. The crude fiber content determination was performed according to the AOAC methods [6]. Two grams of predefatted samples was transferred into a one-liter (1 l) beaker, and then, a sample was digested in a hot plate for 1 h with a mixture of an equal volume of 2.5 M H_2_SO_4_ and 2.5 M NaOH. Then, filtering was performed by moisturizing with a small portion of ethanol. The filtrate was dried in an oven at 100°C until a constant weight was obtained (*W*_1_). Then, the oven-dried samples were again incinerated at 600°C for 3 h in a muffle furnace. Then, the incinerated sample cooled at room temperature and reweighed (*W*_2_).

(4) $$\begin{array}{c} \text{Crude fiber }\left(\%\right)=\frac{\left(W_{1}-W_{2}\right)*100}{S_{\text{W}}}, \end{array}$$

where *W*_1_ is the weight of the porcelain crucible and sample before ashing, *W*_2_ is the weight of the porcelain crucible containing ash, and *W* is the weight of the sample.

*(5) Protein Determination*. The test was performed by the Kjeldahl method of [6]. 0.5 g of sorghum flour sample was weighted into a 50 ml Kjeldahl flask, and 8 ml of concentrated H_2_S0_4_ was added with 2 grams of (copper and potassium sulfate) mixture catalyst. Samples were digested until pure colorless solution was observed. Then, digested samples were distilled by using Kjeldahl distiller, and the distilled steam gas (ammonia) was collected with 25 ml of the mixture of 2% boric acid mixed indicator of bromocresol green plus methyl red. The distilled sample was titrated by 0.1 N HCl until the first appearance of the pink color.

(5) $$\begin{array}{c} \text{Crude protien }\left(\%\right)=\frac{\left(a*b*14*6.25\right)*100}{W}, \end{array}$$

where *a* is the normality of the acid; *b* is the volume of standard acid used (ml), corrected for the blank (i.e., the sample minus the blank); *W* is the sample weight (g); and 6.25 is the conversion factor for protein from %nitrogen.

*(6) Total Carbohydrate*. It is determined as a total carbohydrate by subtracting measured protein, fat, ash, and moisture from 100%. [7].

(6) $$\begin{array}{c} \text{Total carbohydrate }\left(\%\right)=100-\text{Moisture }\left(\%\right)+\text{Protein }\left(\%\right)+\text{Fat }\left(\%\right)+\text{Ash }\left(\%\right). \end{array}$$

*(7) The Gross Food Energy*. The value was estimated by the following equation [8]:

(7) $$\begin{array}{c} \text{Food energy}\left(\frac{\text{kcal}}{\text{g}}\right)=\left(\%\text{TC}-\%\text{CF}\right)\times 4+\left(\%\text{TF}\times 9\right)+\left(\%\text{CP}\times 4\right), \end{array}$$

where TC is the total carbohydrate, CF is the crude fiber, TF is the total fat, and CP is the crude protein.

*(8) Tannin Content*. Tannin was determined by using vanillin-HCL assay methods using a UV spectrophotometer [9] as modified by [10] cereal chemistry. One gram of the sample in a screw cap test tube was measured, and then, 10 ml of 1% HCl in methanol was added to the tube containing the sorghum sample. The sample-containing tube was placed on a mechanical shaker for 24 h at room temperature, and then, the tube was centrifuged at 1000g for 5 minutes. One milliliter (1 ml) of supernatant was taken and mixed with 5 ml of vanillin-HCl reagent in another test tube. Then, the sample was allowed to wait for 20 minutes to complete the reaction, and then, the absorbance of the colored intensity of the sample was measured using a UV-visible spectrophotometer at 500 nm.

(8) $$\begin{array}{c} \text{Tannin }\left(\frac{\text{mg}}{\text{g}}\right)=\frac{\left(A_{\text{s}}-A_{\text{b}}\right)-\left.\text{intercept}\right)}{\text{Slope}*d*W}*10, \end{array}$$

where *A*_s_ is the sample absorbance, *A*_b_ is the blank absorbance, *d* is the density of the solution (0.791 g/ml), *W* is the weight of the sample in gram, and 10 is the aliquot.

*(9) Analysis of the Mineral Composition*. The standard method of [6], wet digestion method, was used; 0.5 grams of flour was taken and digested with 5 ml conc. nitric acid (HNO_3_) and 1 ml conc. perchloric acid (HCLO_4_); then, the digested sample was filtered and made up to 100 ml in a standard flask. The atomic absorption spectrophotometer was used to determine all the minerals (except phosphorus) using appropriate lamps. Phosphorus was determined by UV-visible spectrophotometric method.

#### 2.2.2. Statistical Analysis

The results were subjected to analysis of variance (ANOVA) technique by using the completely randomized design (CRD) method, and all pair-wise comparison tests were used for mean comparison, whereas the least significant difference [11] test was used for mean separation technique at *P* < 0.05.

## 3. Results

### 3.1. Nutritional Compositions

**Figure 1:**
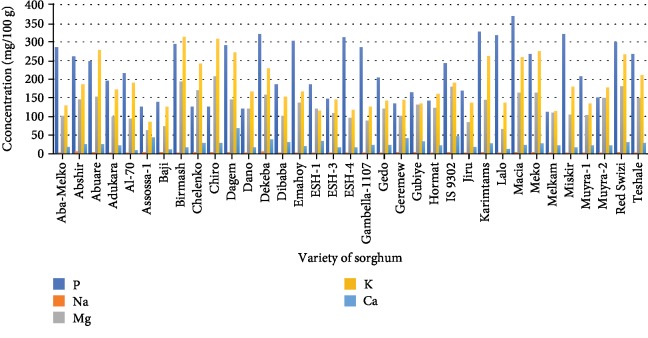
Major mineral contents of sorghum varieties.

**Figure 2:**
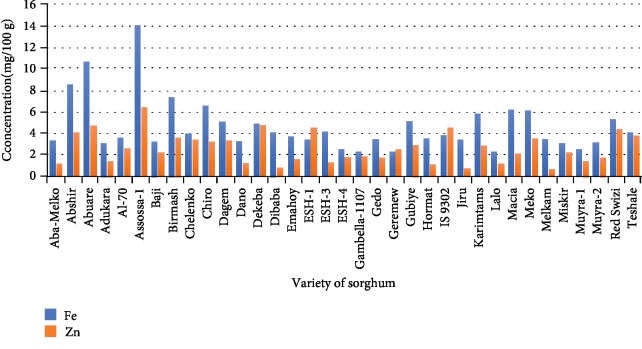
Trace mineral contents of sorghum varieties.

Parameters such as moisture, ash, fat, crude fiber, protein content, total carbohydrate, food energy value, mineral, and tannin are shown in Tables 2 and 3 and Figures 1 and 2. Moisture content ranged from 9.661 to 12.937%, which was significantly different between varieties at *P* ≤ 0.05. The highest value was recorded from a variety of ESH-3 and the lowest from Karimtams. Ash value ranged from 1.119 to 2.294%. The highest ash (total mineral) value was recorded from Gubiye (2.294%), and the lowest value was found from Assossa-1 (1.119). Significant variation was observed at *P* ≤ 0.05 in fat, and its contents varied from 2.481 to 4.596% for the Dagem and Miskir varieties, respectively. Varieties also differed significantly for crude fiber, protein, and carbohydrate with values ranging from 1655 to 8.5865%, 8.201 to 16.476%, and 67.558 to 76.413%, respectively.

### 3.2. Tannin and Energy Value

Varieties varied significantly for tannin content (Table 3). The detectable tannin content of the varieties varied from 1.366 to 3337.2 mg/100 g. The highest value was observed from the varieties Lalo, Dano, and Aba-Melko. The food energy value of sorghum varied from 329.05 to 364.24 kcal. Dagem (364.24 kcal/g) followed by ESH-1 (360.41 kcal/g) and Macia (360.33 kcal/g) resulted in the highest food energy value.

### 3.3. Mineral Composition

Phosphorus (P), zinc (Zn), iron (Fe), calcium (Ca), magnesium (Mg), potassium (K), and sodium (Na) contents are shown in Figures 1 and 2. The highest “P” concentration of 367.965 ppm was obtained from Macia followed by Karimtams with a value of 327.7056 mg/100 g. The lowest concentration of “P” was observed in Melkam varieties (112.554 mg/100 g). Sodium (Na) concentration varied from 2.229 to 6.151 mg/100 g, and the highest concentration was found from Abshir. The highest magnesium (Mg) concentration was found from Chiro whereas the lowest was recorded from Assossa-1 with the respective value of 207.53 mg/100 g and 62.09 mg/100 g. The calcium content was varied from 9.594 mg/100 g to 67.158 mg/100 g. Among the sorghum varieties, the highest “Fe” and “Zn” concentration (14.08 mg/100 and 6.484 mg/100 g, respectively) was found from Assossa-1. “Zn” content varied from 0.698 mg/100 g to 6.484 mg/100 g, and “Fe” content was found between 2.262 and 14.08 mg/100 g.

## 4. Discussion

Sorghum supplies important minerals, vitamins, protein, and micronutrients essential for optimal health, growth, and development [12, 13]. Assessing the nutritional value of sorghum varieties would have a direct impact on the improvement of sorghum for quality breeding and for food product development. The highest moisture content was observed in a variety of ESH-3 (12.937%). Our findings confirm that varieties of sorghum tested were not exposed to deterioration due to mold growth or any damage related to wetness. This could be attributed to a moisture level of less than 15% [14]. The highest ash (total mineral) value was recorded from Gubiye with a value of 2.294%. Different researchers reported variable values of ash for sorghum varieties tested: 1.30 to 3.40 [15], 0.77 to 1.39 [16], 1.43 to 1.92% [17], and 1.51 to 2.06% [18]. Gubiye varieties showed an exceptionally higher ash value than others, and this may be due to variability in agroecology (soil, water, altitude, and climate) at which the sorghum is cultivated [19]. The higher the ash value of the varieties, the higher the total mineral content. Our finding found fat content varied from 2.481 to 4.596% which is in line with reports of [20, 21], and others reported that fat content ranged from 3.44 to 4.90 and 1.38 to 4.50. Moreover, the study in [22] reported that fat value varied from 2.l to 7.6%. Fiber is an important part of carbohydrate; it is a collective name for those that do not readily digest. In this study, the highest value of crude fiber was observed from Abuare (8.5865%) tracked by Lalo (8.1615%) and Gambella-1107 (8.0665%). Accordingly, [15, 23] report fiber content ranging from 1.0 to 3.4% and 0.90 to 4.20%. However, in our study, the results reveal differences as compared to those mentioned in previous research reports. Variability may be attributed to the environment, soil type, genotype variabilities of sorghum, and methods. The varieties with a higher amount of crude fiber might not be good for food consumption because it binds minerals, making them unavailable for absorption [24], and this binding could produce essential mineral imbalance and deficiency. In contrast, it can be useful in products that require hydration, to improve yield and modify texture and viscosity due to its properties of water and oil holding capacity [25]. So, varieties such as Abuare, Lalo, Gambella-1107, and Karimtams are recommended for product development. Carbohydrates provide the majority of energy in the diets of most people. It is a desirable source of energy because it provides easily available energy for the oxidative metabolism of our body. The observed value of carbohydrate ranged from 67.558 to 76.413%. The variety Emahoy, Dibaba, and Assossa-1 had the highest value of 76.413, 76.154, and 76.142 g/100 g, respectively. Results were consistent with the report of [20, 26, 27] who found that the carbohydrate content varied from 67.61 to 73.70, 71.19 to 78.70, and 71.80 to 85.20, respectively. Sorghum is widely used in Ethiopia for human consumption, and it is considered one of the cheap sources of protein. Sorghum has an exogenous factor that influences the digestibility of proteins after cooking. Proteins of cooked sorghum are much less digestible than the proteins of other similarly cooked cereals such as wheat and maize. It is caused by both nonprotein and protein components as reported by [28, 29]. Protein crosslinking is the main factor that affects the low quality of sorghum protein digestibility [29]. Our investigations found that protein varied in the range of 8.201 to 16.476%. The highest protein content was obtained from varieties of Miskir (16.46%) followed by Muyra-2 (16.180%) and ESH-4 (16.178%), whereas the lowest was from Gambella-1107 (8.42%) and Jiru (8.20%). The present findings are in agreement with [2, 30, 31] who reported crude protein content ranging from 3.25 to 14.53%, 7 to 15%, and 10.30 to 14.90%, respectively. Furthermore, [16, 18, 32] reported that sorghum protein content varied from 11.23 to 13.42%, 8.32 to 11.82%, and 9.06 to 18.58%, respectively. The protein content difference that was observed may be attributed to the environment and genotype difference [33]. The varieties Miskir, Muyra-2, and ESH-4 have shown the highest protein content than the others. Identified sorghum varieties should be considered for food product development to combat protein malnutrition and for the breeders to improve sorghum nutritional quality. In this study, the tannin content of the sorghum varieties was also included. Tannin is the most abundant antinutritional factor in sorghum [34]. Multiple phenolic hydroxyl groups of tannins may form stable complexes with protein, metal ions, and other macromolecules like polysaccharides [35, 36] and will reduce the digestibility of the proteins and the availability of the nutrients in the gut. Interestingly, the highest tannin concentration was found in the variety of Lalo, with the average value of 3337.2 mg/100 g. Different tannin concentration of sorghum was previously reported by [37, 38] who found 10 mg/100 g to 351 mg/100 g and 0.021 to 0.681%. Besides, [39] in Tanzania reported a range of tannin content for 12 sorghum varieties which was found to be 2.18% to 5.76%. Food crops having high antinutritional content may not be good for food consumption, due to its negative impact on nutrient availability and digestibility unless processed very well. Besides, the sensory of the food product may not be accepted due to bitterness characteristics. So, the varieties Abshir, ESH-1, Esh-3, Gambela-1170, Macia, Meko-1, Melkam, and Teshale had very low tannin content and might be the best for food product making quality in both sensory attributes and mineral-protein bioavailability. In the developing country which has a high population growth rate, deficiency of mineral nutrient and protein source food is a critical problem. Mineral nutrients play an important role in the development of the human body [40]. In this study, important micronutrient mineral contents were investigated including phosphorus (P), magnesium (Mg), potassium (K), and sodium (Na). “P” concentration ranged from 112.554 mg/100 g (Melkam) to 367.965 mg/100 g (Macia). The results of “P” in this experiment are consistent with reports of [41, 42] who reported “P” concentration values ranging from 1498 mg kg^−1^ to 3787.25 mg kg^−1^ and 2505.83 mg kg^–1^ to 3453 mg kg^–1^. Some results of “P” were found below the reports, and it may arise from the soil type of the environment and the variety variability. Sodium “Na” concentration varied from 2.229 to 6.151 mg/100 g which is in agreement with the report of [41] that reported “Na” concentration varying from 11.5 to 54.38 mg/kg, 6.3 to 7.0 mg/100 g, and 5.83 to 6.18 mg/100 g, respectively. “Mg” concentration of this study varied between 207.53 mg/100 g and 62.09 mg/100 g. The result of “Mg” is fully in agreement with [31] who found a concentration of “Mg” ranging from 65.00 to 375.26 mg/100 g and 750 mg/kg to 1506.3 mg/kg. Calcium is a mineral necessary to build and maintain strong bones and teeth. The average calcium makes up about 2.3% of a person's body weight. The highest “Ca” content was obtained from Dagem (67.158 mg/100 g) whereas the lowest was found from Al-70 (9.594 mg/100 g) as shown in Figure 1. Dagem showed higher calcium value than other tested varieties. Accordingly, [41] report from 31 landraces of sorghum the highest and the lowest “Ca” concentration varying from 477.50 mg/kg to 207.50 mg/kg, respectively, and it is comparable to our findings. On the other hand, from the report of [31], “Ca” concentration for the popular varieties of sorghum ranged from 7.96 mg/100 g to 38.78 mg/100 g, and for the 91 germ plasma concentration, “Ca” varied from 2.10 mg/100 g to 255.26 mg/100 g. Iron and zinc are essential micronutrients in the body. Iron (Fe) is a core element in the synthesis of hemoglobin and myoglobin [43]. Its deficiency strongly relates to anemia, mental disorder, immunity problems [44] children's cognitive ability, poor pregnancy quality, and lower working capacity in adults [45]. Iron deficiency is mainly caused by low intake of heme iron and vitamin C. Iron contributes to the formation of heme enzymes and other Fe-containing enzymes that are important for energy production, immune defense, and thyroid function whereas zinc (Zn) is contributing as a good reducing agent. It can easily form a complex with other compounds including carbonates, phosphates, sulfates, oxalates, and phytates. An important function of zinc is an essential cofactor of more than 70 enzymes [46]. Its deficiency is ranked in the top five risk factors of disease and death in developing countries [47]. Zinc deficiency caused dermatitis, growth retardation, diarrhea, mental disturbances, and recurrent infections [48]. This deficiency generally relates to diet consumption with low zinc or high in phytates. Iron and zinc deficiency, a common nutritional disorder, is a substantial factor in health problems all over the world especially in the developing and low-income countries [48]. In the current research system of sorghum in Ethiopia, there is a need for further research to improve the iron and zinc contents of sorghum through modernizing breeding in addition to protein because of their high advantages in our body system in the processes of creating a healthy society. Due to micronutrient deficiency in food, interventions are targeting on iron and zinc deficiencies including dietary variation, food supplementation, (bio)fortification, and improvement in bioaccessibility and bioavailability. The finding shows that the highest “Fe” and “Zn” concentration is from Assossa-1 with the respective values of 14.08 mg/100 g and 6.484 mg/100 g. As reported in this study of [49] who found that the “Fe” content ranged from 4.70 to 14.05 mg per 100 g, the current study findings are also in line with this research report. Also, in [39], the iron concentration study on 12 varieties of sorghum from Tanzania showed iron content in the range of 5.50 mg/100 g to 182 mg/100 g. Zinc concentration varied from 0.698 mg/100 g to 6.484 mg/100 g. This is in accordance with reports of [22, 31, 50] but higher than the report of [41, 51]. In general, for iron, varieties Abshir, Abuare, Assossa-1, Birmash, Chiro, Macia, and Meko-1, whereas for Zinc, Abshir, Abuare, Assossa-1, Dekeba, ESH-1, Red Swazi, and Teshale, need to get attention for micronutrient enrichment for both food product development and for improving sorghum grain micronutrient quality. Accordingly, in the [52] research report, the grain of sorghum which has greater than 5 mg/100 g and 3.70 mg/100 g of Fe and Zn, respectively, has been recommended as the potential for grain micronutrient enrichment. The majority of Asian and African people consume sorghum as a whole grain. Although sorghum bran contains iron [53] phytate and sometimes tannins, depending on the cultivar, they reduce the availability of iron [54]. However, several studies have shown that in vitro bioavailability of iron and zinc improved by soaking and germination processing methods [55]. This research report reveals that both “K” and “P” were recorded in a dominant mineral in sorghum followed by “Mg,” and it has been stated by [56]. In general, the mineral contents of sorghum in this study were varied between the varieties, and it may arise from genotype differences, mineral concentration in the soil, environmental factors, and development period of the plants influence the final grain composition [41].

### 4.1. Pearson's Correlations

The tannin was negatively correlated with ash, fat, moisture, and protein as shown in Table 4, but the correlation is not significant except protein. Protein is significantly negatively correlated with tannin (*P* value, 0.0312). The protein of cereals is less digestible than animal proteins, due to the presence of different antinutritional factors. Tannin-protein interaction in sorghum involves hydrogen bonding and hydrophobic interactions whereby tannins are capable of binding and precipitating at least 12 times their weight of protein [57]. In the case of sorghum, protein is less digestible than that of other cereals, thus reducing the bioavailability of the protein. This poor digestibility is due to extensive polymerization of kafirins upon cooking and the presence of tannins in certain sorghum lines [28].

## 5. Conclusion

In our investigation, we found that varieties of sorghum had shown differences in all tested parameters. Differences were attributed to the genetical difference of sorghum varieties, environmental conditions, and soil type. Varieties of sorghum have their nutritional quality. However, the researchers recommend that Assossa-1, Abuare, Abshir, Meko-1, Red Swazi, and Birmash varieties of sorghum will be a good source of iron and zinc micronutrient whereas Miskir, Muyra-2, ESH-4, and Karimtams for protein content. To summarize, Abshir, ESH-1, Meko-1, Red Swazi, and Karimtams contain a well-adjusted protein, mineral (Fe, Zn, and Ca), and less tannin content. So, the food industries, consumers, and breeders need to be considering those selected varieties for food product development and for generating quality sorghum grain for combatting malnutrition in our country. Thus, in the light of scientific data of the present investigation, it may be concluded that evaluating the nutritional values of different varieties of sorghum is very important for designing and developing products of higher nutritional quality for ensuring food quality and helping the sorghum breeders for further improvement of varieties based on the nutrition aspect. In this study, the researchers recommend that low-income communities better use those selected varieties of sorghum to prevent the prevalence of childhood undernutrition since economic and contextual factors are strong determinants of child nutritional status [58–60]. The researchers suggest that further study on the functional property of the flour, phytate, protein digestibility, and mineral bioavailability for selected sorghum varieties is needed.

## Figures and Tables

**Table 1:** General physical characteristics of sorghum varieties.

Varieties	Potential growth area	Purpose of release	Seed color
Abshir	Dry lowlands (<1600 masl)	Striga resistance	White
Gubiye			
Abuare		Early	White
Dekeba			
Miskir			
Hormat			
ESH-1		High yielder	White
ESH-3			
Gambella-1107			
Gedo			
Red Swazi		For malt	Brown
Macia			White
Teshale			White
Meko-1		Early, quality	
Melkam			

Chiro	Highlands (>1900 masl)	High yielder, sweet stalk	Brown
Al-70		High yielder, quality	White
Dibaba		High yielder	Brown
Chelenko			
Muyra-1			
Muyra-2			White

Dagem	Intermediate altitudes (1600)	High yielder	Brown
Baji			
Birmash			
Geremew			
IS-9302			
Aba-Melko		High yielder, disease resistance	
Lalo			
Dano			Orange

Emahoy	Wet lowlands (<1600 masl)	High yielder, disease resistance	Brown
Adukara			Red

**Table 2:** Nutritional compositions of sorghum varieties (g/100 g).

No.	Varieties	Moisture (%)	Ash (%)	Fat (%)	Fiber (%)	Protein (%)	CHO (%)
1	Aba-Melko	10.411^g-j^	1.5100^b-e^	3.5625^e-g^	5.6010^kl^	8.9810^m^	75.536^b-d^
2	Abshir	10.984^cd^	1.6295^b-e^	4.0005^cd^	6.9995^ef^	12.716^d^	70.669^qr^
3	Abuare	10.244^i-l^	1.6010^b-e^	3.2715^g-k^	8.5865^a^	11.541^f^	73.343^i-l^
4	Adukara	10.429^f-j^	1.7140^b-e^	3.3955^g-k^	2.1655^u^	9.0180^m^	75.444^b-e^
5	Al-70	11.534^b^	1.6005^b-e^	3.9240^cd^	6.5915^g^	11.079^gh^	71.863^no^
6	Assossa-1	10.283^h-l^	1.1195^f^	3.2945^g-k^	3.1150^qr^	9.1610^m^	76.142^ab^
7	Baji	11.073^c^	1.4875^c-e^	3.5120^e-i^	6.0240^h-j^	9.8240^kl^	74.103^g-i^
8	Birmash	10.806^c-f^	1.8410^b^	3.5405^e-h^	3.4415^o-q^	10.870^g-i^	72.942^j-l^
9	Chelenko	9.9805^k-n^	1.6450^b-e^	4.5360^ab^	5.4420^l^	10.197^jk^	73.642^h-j^
10	Chiro	11.693^b^	1.7405^b-d^	3.8305^de^	6.4280^g^	11.644^f^	71.092^pq^
11	Dagem	10.637^d-h^	1.4315^d-f^	4.5960^a^	2.6160^st^	10.675^hi^	72.660^k-m^
12	Dano	10.251^i-l^	1.7365^b-d^	2.5705^no^	4.3435^m^	11.104^g^	74.338^f-h^
13	Dekeba	10.555^e-i^	1.6215^b-e^	2.5345^no^	6.5725^g^	9.9665^kl^	75.322^c-e^
14	Dibaba	10.563^e-i^	1.4605^c-f^	2.8040^m-o^	4.0055^mn^	9.0190^m^	76.154^ab^
15	Emahoy	10.276^h-l^	1.4720^c-e^	2.7530^no^	3.8435^no^	9.0865^m^	76.413^a^
16	ESH-1	10.602^d-i^	1.6140^b-e^	4.3420^ab^	3.1080^qr^	13.572^c^	69.870^s^
17	ESH-3	12.937^a^	1.6775^b-e^	3.1370^k-m^	7.0445^ef^	13.648^c^	68.601^tu^
18	ESH-4	11.482^b^	1.5055^b-e^	3.2780^g-k^	2.4015^tu^	16.178^ab^	67.558^v^
19	Gambella-1107	10.674^d-g^	1.4255^d-f^	3.4810^f-j^	8.0665^bc^	8.4250^n^	75.995^a-c^
20	Gedo	9.9690^l-n^	1.8015^bc^	2.7205^no^	3.3490^pq^	10.514^ij^	74.995^d-f^
21	Geremew	10.453^f-j^	1.7670^b-d^	4.2100^bc^	7.3385^de^	10.959^gh^	72.612^l-n^
22	Gubiye	10.730^c-g^	2.2940^a^	3.2905^g-k^	7.6890^cd^	13.718^c^	69.966^rs^
23	Hormat	10.476^f-j^	1.5595^b-e^	2.6165^no^	6.3535^g-j^	12.284^e^	73.063^j-l^
24	IS-9302	10.652^d-h^	1.7100^b-e^	3.5205^e-i^	6.4655^g^	10.697^g-i^	73.421^i-k^
25	Jiru	11.488^b^	1.4900^c-e^	3.2150^h-k^	5.3660^l^	8.2015^n^	75.606^b-d^
26	Karimtams	9.6605^n^	1.6140^b-e^	2.8440^l-n^	7.8385^bc^	15.913^b^	69.968^rs^
27	Lalo	9.7610^mn^	1.4860^c-e^	3.2000^i-k^	8.1615^b^	9.5955^l^	75.957^a-c^
28	Macia	10.398^g-j^	1.4405^d-f^	3.8155^d-f^	2.8480^rs^	12.447^de^	71.899^m-o^
29	Meko-1	10.110^j-m^	1.6615^b-e^	3.8005^d-f^	3.6010^op^	12.610^de^	71.819^op^
30	Melkam	11.724^b^	1.6695^b-e^	3.1550^j-l^	6.3870^gh^	9.5965^l^	73.855^hi^
31	Miskir	9.9590^l-n^	1.7295^b-e^	2.4810^o^	6.4735^g^	16.476^a^	69.355^st^
32	Muyra-1	10.473^f-j^	1.5140^b-e^	3.5705^e-g^	5.9830^i-k^	9.7425^l^	74.701^e-g^
33	Muyra-2	10.355^g-k^	1.5605^b-e^	3.8220^de^	6.6415^fg^	16.180^ab^	68.082^uv^
34	Red Swazi	10.956^cd^	1.6370^b-e^	3.3905^g-k^	7.0300^ef^	12.540^de^	71.477^op^
35	Teshale	10.880^c-e^	1.3885^e-f^	3.5735^e-g^	5.6805^j-l^	11.072^gh^	73.085^j-l^
	SEM	0.134	0.119	0.117	0.140	0.145	0.2674
	LSD at *P* < 0.05	0.3834	0.3419	0.3351	0.4031	0.4169	0.7676

Means with different superscripts are significantly different at *P* < 0.05.

**Table 3:** Tannin content of sorghum (mg/100 g) and food energy (kcal/g) in dry base.

No.	Varieties	Tannin	FE
1	Aba-Melko	2418.9^c^	347.73^gh^
2	Abshir	BDL	341.55^j-m^
3	Abuare	15.752^o^	334.63^pq^
4	Adukara	488.27^g^	359.74^b^
5	Al-70	4.7050^op^	340.72^k-n^
6	Assossa-1	787.65^e^	358.40^b^
7	Baji	120.80^n^	343.22^i-k^
8	Birmash	158.75^l^	353.35^de^
9	Chelenko	12.523^o^	354.41^c-e^
10	Chiro	167.77^j-l^	339.71^l-o^
11	Dagem	120.95^n^	364.24^a^
12	Dano	2474.7^b^	347.53^gh^
13	Dekeba	4.9095^op^	337.67^n-p^
14	Dibaba	176.29^j^	349.91^fg^
15	Emahoy	755.77^f^	351.40^ef^
16	ESH-1	BDL	360.41^b^
17	ESH-3	BDL	329.05^r^
18	ESH-4	137.25^m^	354.84^cd^
19	Gambella-1107	BDL	336.74^o-q^
20	Gedo	5.0075^op^	353.12^d-f^
21	Geremew	160.65^kl^	342.82^i-l^
22	Gubiye	166.52^j-l^	333.60^q^
23	Hormat	6.5560^op^	339.52^l-o^
24	IS-9302	171.27^jk^	342.29^j-l^
25	Jiru	253.24^h^	342.70^i-l^
26	Karimtams	13.104^o^	337.77^n-p^
27	Lalo	3337.2^a^	338.37^m-o^
28	Macia	BDL	360.33^b^
29	Meko-1	BDL	357.51^bc^
30	Melkam	BDL	336.65^o-q^
31	Miskir	1.3660^p^	339.76^l-o^
32	Muyra-1	207.37^i^	345.97^hi^
33	Muyra-2	13.745^o^	344.88^h-j^
34	Red Swazi	902.48^d^	338.46^m-o^
35	Teshale	BDL	346.07^hi^
	SEM	3.8134	
	LSD	11.065	

Means with different superscripts are significantly different at *P* < 0.05. BDL: below detection limit.

**Table 4:** Pearson correlations of proximate compositions of sorghum.

	Ash	Fat	Fiber	Moisture	Protein
Fat	-0.0472				
*P* value	0.7349				
Fiber	0.2336	-0.0148			
	0.0891	0.9157			
Moisture	0.0770	0.2682	-0.1220		
	0.5799	0.0499	0.3795		
Protein	0.2580	-0.1173	0.2658	-0.0618	
	0.0597	0.3981	0.0520	0.6572	
Tannin	-0.1448	-0.1460	0.0494	-0.2665	-0.2935^∗∗^
	0.2963	0.2922	0.7226	0.0514	0.0312^∗∗^

^∗∗^Significantly correlated.

## Data Availability

We assure that the data availability is a must and all the results are available.

## References

[1] Zhao Z.-Y., Che P., Glassman K., Albertsen M., Zhao Z. Y., Dahlberg J. (2019). Nutritionally enhanced sorghum for the arid and semiarid tropical areas of Africa. *Sorghum*.

[2] Dicko M. H., Gruppen H., Traoré A. S., Voragen A. G., van Berkel W. J. (2006). Sorghum grain as human food in Africa: relevance of content of starch and amylase activities. *African Journal of Biotechnology*.

[3] Kumar S. V., Sajeevkumar V., George J., Kumar S. (2017). Enhancing properties of polyvinyl alcohol film using sorghum starch nanocrystals – a cost effective filler from natural source. *Defence Life Science Journal*.

[4] Spielman D. J., Kelemwork D., Alemu D. (2012). Seed, fertilizer, and agricultural extension in Ethiopia. *Food and agriculture in Ethiopia: Progress and policy challenges*.

[5] Boudries N., Belhaneche N., Nadjemi B. (2009). Physicochemical and functional properties of starches from sorghum cultivated in the Sahara of Algeria. *Carbohydrate Polymers*.

[6] Horwitz W. (2000). *Official Methods of Analysis of AOAC International 17th Edition*.

[7] Pearson D. (1976). The Dictionary of Nutrition and Food Technology. *Butterworth International Conference on Appplied*.

[8] Edeoga H., Okwu D., Mbaebie B. (2003). Minerals and nutritive value of some Nigerian medicinal plants. *Journal of Medicinal and Aromatic Plant Science*.

[9] Burns R. E. (1971). Method for estimation of tannin in grain sorghum 1. *Agronomy Journal*.

[10] Maxson E., Rooney L. (1972). Evaluation of methods for tannin analysis in sorghum grain. *Cereal Chemistry*.

[11] Steel R. G. D., Torrie J. H. (1960). *Principles and Procedures of Statistics*.

[12] Chan S. S. L., Ferguson E. L., Bailey K., Fahmida U., Harper T. B., Gibson R. S. (2007). The concentrations of iron, calcium, zinc and phytate in cereals and legumes habitually consumed by infants living in East Lombok, Indonesia. *Journal of Food Composition and Analysis*.

[13] Salgueiro M. J., Zubillaga M. B., Lysionek A. E., Caro R. A., Weill R., Boccio J. R. (2002). The role of zinc in the growth and development of children. *Nutrition*.

[14] Onimawo I. A., Oteno F., Orokpo G., Akubor P. I. (2003). Physicochemical and nutrient evaluation of African bush mango (Irvingia gabonensis) seeds and pulp. *Plant Foods for Human Nutrition*.

[15] Moharram Y., Youssef A. (1995). Sorghum grain and quality of its edible products. *Developments in Food Science*.

[16] Chung I.-M., Kim E. H., Yeo M. A., Kim S. J., Seo M.–. C., Moon H. I. (2011). Antidiabetic effects of three Korean sorghum phenolic extracts in normal and streptozotocin-induced diabetic rats. *Food Research International*.

[17] Pontieri P., Di Fiore R., Troisi J. (2012). Chemical composition and fatty acid content of white food sorghums grown in different environments. *Maydica*.

[18] Hamad R. M. E. (2006). *Preliminary studies on the popping characteristics of sorghum grains*.

[19] Bryden W., Selle P. H., Cadogan D. J. (2009). *A review of the nutritive value of sorghum for broilers, Publication No. 09/077*.

[20] Okoh P. N., Obilana A. T., Njoku P. C., Aduku A. O. (1982). Proximate analysis, amino acid composition and tannin content of improved Nigerian sorghum varieties and their potential in poultry feeds. *Animal Feed Science and Technology*.

[21] Buffo R. A., Weller C. L., Parkhurst A. M. (1998). Relationships among grain sorghum quality factors. *Cereal Chemistry*.

[22] Jambunathan R. (1980). Improvement of the nutritional quality of sorghum and pearl millet. *Food and Nutrition Bulletin*.

[23] Jambunathan R., Singh U., Subramanian V. (1981). *Grain Quality of Sorghum, Pearl Millet, Pigeon-pea, and Chick-pea*.

[24] Nilka de Oliveira M., Ponte Freitas A., Urano Carvalho A. (2009). Nutritive and non-nutritive attributes of washed-up seaweeds from the coast of Ceara, Brazil. *Food Chemistry*.

[25] Elleuch M., Bedigian D., Roiseux O., Besbes S., Blecker C., Attia H. (2011). Dietary fibre and fibre-rich by-products of food processing: characterisation, technological functionality and commercial applications: a review. *Food Chemistry*.

[26] Desai B. B., Inamdar D. G., Chavan U. D., Naik R. M. (1984). Proximate composition and protein-fractions of some promising sorghum cultivars. *Journal of Maharashtra Agricultural Universities*.

[27] Neucere N. J., Sumrell G. (1980). Chemical composition of different varieties of grain sorghum. *Journal of Agricultural and Food Chemistry*.

[28] Duodu K. G., Taylor J. R. N., Belton P. S., Hamaker B. R. (2003). Factors affecting sorghum protein digestibility. *Journal of Cereal Science*.

[29] Duodu K. G., Nunes A., Delgadillo I. (2002). Effect of grain structure and cooking on sorghum and maize in vitro protein digestibility. *Journal of Cereal Science*.

[30] Johnson W. B., Ratnayake W. S., Jackson D. S. (2010). Factors affecting the alkaline cooking performance of selected corn and sorghum hybrids. *Cereal Chemistry*.

[31] Badigannavar A., Girish G., Ramachandran V., Ganapathi T. R. (2016). Genotypic variation for seed protein and mineral content among post-rainy season-grown sorghum genotypes. *The Crop Journal*.

[32] Gu-Ayebeafo Okrah S. (2008). *Screening of six local sorghum varieties for their malting and brewing qualities*.

[33] Deosthale Y., Nagarajan V., Rao K. V. (1972). Some factors influencing the nutrient composition of sorghum grain. *Indian Journal of Agricultural Sciences*.

[34] Selle P. H., Cadogan D. J., Li X., Bryden W. L. (2010). Implications of sorghum in broiler chicken nutrition. *Animal Feed Science and Technology*.

[35] Choct M., Hughes R. J. (1999). Chemical and physical characteristics of grains related to variability in energy and amino acid availability in poultry. *Australian Journal of Agricultural Research*.

[36] Kondo M., Kita K., Yokota H.-o. (2007). Ensiled or oven-dried green tea by-product as protein feedstuffs: effects of tannin on nutritive value in goats. *Asian-Australasian Journal of Animal Sciences*.

[37] Radhakrishnan M. R., Sivaprasad J. (1980). Tannin content of sorghum varieties and their role in iron bioavailability. *Journal of Agricultural and Food Chemistry*.

[38] Sedghi M., Golian A., Soleimani-Roodi P., Ahmadi A., Aami-Azghadi M. (2012). Relationship between color and tannin content in sorghum grain: application of image analysis and artificial neural network. *Brazilian Journal of Poultry Science*.

[39] Kaijage J., Mutayoba S., Katule A. (2014). Chemical composition and nutritive value of Tanzanian grain sorghum varieties. *Livestock Research for Rural Development*.

[40] Ng’uni D., Geleta M., Hofvander P., Fatih M., Bryngelsson T. (2012). Comparative genetic diversity and nutritional quality variation among some important Southern African sorghum accessions [‘*Sorghum bicolor*’(L.) Moench]. *Australian Journal of Crop Science*.

[41] Shegro A., Shargie N. G., van Biljon A., Labuschagne M. T. (2012). Diversity in starch, protein and mineral composition of sorghum landrace accessions from Ethiopia. *Journal of Crop Science and Biotechnology*.

[42] Gerrano A., Labuschagne M. T., van Biljon A., Shargie N. G. (2016). Quantification of mineral composition and total protein content in sorghum [*Sorghum bicolor* (L.) Moench] genotypes. *Cereal Research Communications*.

[43] Nkhata S. G., Ustunol Z., Menevseoglu A. (2013). *Iron fortification of yogurt and pasteurized milk, [M.S. thesis]*.

[44] Gaucheron F. (2000). Iron fortification in dairy industry. *Trends in Food Science & Technology*.

[45] Derbyshire E., Brennan C. S., Li W., Bokhari F. (2010). Iron deficiency–is there a role for the food industry?. *International Journal of Food Science & Technology*.

[46] Berdanier C. D., Berdanier L. A. (2015). *Advanced nutrition: macronutrients, micronutrients, and metabolism*.

[47] World Health Organization (2002). *The world health report 2002: reducing risks, promoting healthy life*.

[48] Dary O., Hurrell R. (2006). *Guidelines on Food Fortification with Micronutrients*.

[49] Deosthale Y., Belavady B. (1978). Mineral and trace element composition of sorghum grain: effect of variety, location and application of the nitrogen fertilizer. *Indian Journal of Nutrition and Dietetics*.

[50] Kayodé A. P., Linnemann A. R., Hounhouigan J. D., Nout M. J., van Boekel M. (2006). Genetic and environmental impact on iron, zinc, and phytate in food sorghum grown in Benin. *Journal of Agricultural and Food Chemistry*.

[51] Patekar S., Moreand D., Hashmi S. (2017). Studies on physico-chemical properties and minerals content from different sorghum genotypes. *Journal of Pharmacognosy and Phytochemistry*.

[52] Ashok Kumar A., Reddy B. V., Ramaiah B., Sanjana Reddy P., Sahrawat K. L., Upadhyaya H. D. (2009). Genetic variability and plant character association of grain Fe and Zn in selected core collection accessions of sorghum germplasm and breeding lines. *Journal of SAT Agricultural Research*.

[53] Mahgoub S. E., Elhag S. A. (1998). Effect of milling, soaking, malting, heat-treatment and fermentation on phytate level of four Sudanese sorghum cultivars. *Food Chemistry*.

[54] Hunt J. R. (2003). Bioavailability of iron, zinc, and other trace minerals from vegetarian diets. *The American Journal of Clinical Nutrition*.

[55] Afify A. E.-M. M., El-Beltagi H. S., Abd El-Salam S. M., Omran A. A. (2011). Bioavailability of iron, zinc, phytate and phytase activity during soaking and germination of white sorghum varieties. *PLoS One*.

[56] Khalil J., Sawaya W. N., Safi W. J., Al-Mohammad H. M. (1984). Chemical composition and nutritional quality of sorghum flour and bread. *Plant Foods for Human Nutrition*.

[57] Butler L. G., Riedl D. J., Lebryk D. G., Blytt H. J. (1984). Interaction of proteins with sorghum tannin: mechanism, specificity and significance. *Journal of the American Oil Chemists’ Society*.

[58] Hatløy A., Hallund J., Diarra M. M., Oshaug A. (2000). Food variety, socioeconomic status and nutritional status in urban and rural areas in Koutiala (Mali). *Public Health Nutrition*.

[59] Agee M. D. (2010). Reducing child malnutrition in Nigeria: combined effects of income growth and provision of information about mothers’ access to health care services. *Social Science & Medicine*.

[60] Kanjilal B., Mazumdar P., Mukherjee M., Rahman M. H. (2010). Nutritional status of children in India: household socio-economic condition as the contextual determinant. *International Journal for Equity in Health*.

